# Fungal Extract of *Lasiodiplodia pseudotheobromae* IBRL OS-64 Inhibits the Growth of Skin Pathogenic Bacterium and Attenuates Biofilms of Methicillin-Resistant *Staphylococcus aureus*

**DOI:** 10.21315/mjms2021.28.4.4

**Published:** 2021-08-26

**Authors:** Mohd Taufiq Mat Jalil, Darah Ibrahim

**Affiliations:** 1 School of Biology, Faculty of Applied Sciences, Universiti Teknologi MARA, Shah Alam, Selangor, Malaysia; 2 Industrial Biotechnology Research Laboratory, School of Biological Sciences, Universiti Sains Malaysia, Pulau Pinang, Malaysia

**Keywords:** L. pseudotheobromae IBRL OS-64, MRSA, antibiofilm activity, biofilm formation, semi-quantitative adherence assays

## Abstract

**Background:**

The emergence of multidrug-resistant pathogens associated with biofilm formation can cause life-threatening infections to humans. Therefore, the present study aims to evaluate the effects of the fungal extract of *Lasiodiplodia pseudotheobromae* (*L. pseudotheobromae*) Industrial Biotechnology Research Laboratory (IBRL) OS-64 on bacterial cells and the biofilm formation of methicillin-resistant *Staphylococcus aureus* (MRSA).

**Methods:**

Broth microdilution and semi-quantitative adherence assays were conducted to determine the anti-biofilm activity of the fungal extract. Light and scanning electron microscopy (SEM) analyses were performed to observe the effect of the fungal extract on biofilm formation by MRSA.

**Results:**

The transmission electron microscopy (TEM) microphotographs showed that the bacterial cells were severely damaged upon 24 h exposure to the extract and displayed several symptoms such as cell shrinkage and breakage. Meanwhile, results from the antibiofilm study indicated the extract attenuated the initial and preformed biofilms of MRSA by 80.82% and 61.39%, respectively. The initial biofilm was more sensitive to the extract compared to the pre-formed biofilm, as evidenced by the light microscopy and SEM observations that demonstrated more severe bacterial cell damage on the initial biofilms compared to pre-formed biofilms.

**Conclusion:**

The ethyl acetate extract of *L. pseudotheobromae* IBRL OS-64 significantly inhibited bacterial cells growth and eliminated biofilm formation by MRSA.

## Introduction

Staphylococci are Gram-positive, non-motile and non-spore forming facultative anaerobe bacteria that are capable of growing by fermentation or aerobic respiration. They are characterised by individual cocci which divide in more than one plane to form grape-like clusters. *Staphylococcus aureus* (*S. aureus)* and *S. epidermis* are the most widely characterised and studied strains amongst this genus (1). *S. aureus* is a major common human pathogen that can cause a wide variety of clinical manifestations ranging from minor skin infections to severe systemic diseases, including pneumonia and septicaemia. Fortunately, the introduction of antibiotics has dramatically improved the condition of infected patients as the ability of antibiotics to combat infection has significantly reduced the number of incidences of the bacterial infection, thus, improving the quality of life of patients, increasing life expectancy, reducing childhood mortality and saving human lives (2).

Unfortunately, the discovery and increasingly widespread use, including the misuse of antibiotics have led to the emergence of antibiotic-resistant strains and resulted in more reported cases of infections by pathogenic microorganisms that fail to respond to traditional antimicrobial treatments. Methicillin-resistant *S. aureus* (MRSA) is an example of antibiotic-resistant strains that have spread worldwide, causing global human health concerns. This strain is widely prevalent worldwide with infection rates exceeding 50% reported in Asia, Malta, North and South Africa (3). According to Chambers (4), MRSA is resistant to β-lactam antibacterial agents due to the expression of additional penicillin-binding protein (PBP2a) acquired from another species which is resistant to the actions of the antibiotic. Furthermore, the use of different types of antibiotics in clinical treatments over the years has led to the appearance of multi-resistant MRSA strains as a result of the acquisition and accumulation of antibiotic resistant-conferring genes as well as mutation in a gene coding for target proteins (5).

*S. aureus* strains can form surface-adherent communities called biofilm, which enables them to survive under stressful environments and conditions, including nutrient limitations, heat shock, and the actions of antibiotics and immune responses. Nowadays, biofilm-forming capacity is recognised as an important virulence determinant in the development of staphylococcal device-related infections (6). Similarly, Yaghoobi et al. (7) stated that the virulence of the *S. aureus* strains is due to their adherence and invasion strategies which are associated with the formation of biofilms capable of acting as a barrier against antibiotics diffusion. The increment of multidrug-resistant strains is a global health problem and this has spurred many researchers to search for effective antibacterial and antibiofilm drugs to combat the virulence of MRSA. There are several strategies to eradicate biofilm formation including inhibiting the adhesion of bacteria to living or non-living surfaces at the initial stage, which could then reduce the development and establishment of a biofilm (6), disruption of biofilm architecture during the maturation process (8), and antipathogenic or signal interference by inhibiting quorum sensing (QS) and expression virulence factors of *S. aureus* (9).

For centuries, endophytic fungi isolated from medicinal plants have been utilised worldwide for their bioactive compounds with pharmaceutical potentials. An endophytic fungus, *Lasiodiplodia pseudotheobromae* (*L. pseudotheobromae*) has been reported to possess antibacterial properties against pathogenic bacteria, including *S. aureus* and MRSA (10). Therefore, the present study was designed to investigate the antibiofilm properties of the ethyl acetate extract of *L. pseudotheobromae* Industrial Biotechnology Research Laboratory (IBRL) OS-64 against biofilm formation of MRSA.

## Methods

### Maintenance of Endophytic Fungus and Test Bacterium Cultures

The endophytic fungus, *L. pseudotheobromae* IBRL OS-64 and MRSA ATCC 33591 were provided by the IBRL, School of Biological Sciences, Universiti Sains Malaysia, Pulau Pinang, Malaysia. The fungal isolate was cultured on potato dextrose agar (PDA) nourished with powdered host plant (2 g/L), the bacterial culture was grown on nutrient agar (NA) and the cultures were incubated at 30 °C and 37 °C, respectively. Both fungal isolate and test bacterium were stored at 4 °C before use and subcultured regularly on sterile fresh media to ensure their survivability.

### Cultivation and Extraction

The cultivation of the fungus was performed in submerged fermentation using yeast extract sucrose (YES) broth as a cultivation medium and the extraction of the culture was carried out according to Taufiq and Darah (11).

### Bacterial Suspension

The inoculum suspension of MRSA ATCC 33591 was prepared by inoculating aseptically five single colonies from 24 h-old cultures into 5 mL of 0.85% sterile physiological saline (w/v). The inoculum size was standardised by matching its turbidity to the 0.5 McFarland standards, which was equivalent to 1 × 10^8^ CFU/mL.

### Congo Red Agar Assay

Congo red agar (CRA) assay was prepared according to the method described by Torlak et al. (13). The plates were incubated for 24 h at 37 °C. After 24 h of incubation, the plates were observed and data was recorded.

### Inoculum Preparation for Anti-Biofilm Assay

The inoculum of MRSA ATCC 33591 was prepared according to Taufiq and Darah (14). The bacterial strain was cultured in Mueller-Hinton agar (MHA) and incubated for 24 h at 37 °C. After 24 h of incubation, five colonies of MRSA ATCC 33591 were then inoculated into a 250 mL Erlenmeyer flask containing 50 mL Mueller-Hinton broth (MHB), incubated at 37 °C for 24 h and agitated at 150 rpm. The bacterial broth culture was then centrifuged at 4000 rpm, 4 °C for 30 min to separate the supernatant and the pellet. The cell pellet was collected and resuspended with 10 mL MHB and the supernatant was discarded. The cell densities of the suspensions were adjusted to an optical density (OD) of 0.15 at 600 nm by dilution with fresh MHB.

### Semi-Quantitative Adherence Assay

The anti-biofilm activity of MRSA ATCC 33591 was performed using a semi-quantitative adherence assay method by Taufiq and Darah (14). The plates were air-dried overnight and the OD of each well was measured at 570 nm and recorded.

### Inhibition Assay of the Initial and Pre-Formed Biofilm

The inhibition assay of the initial and preformed biofilm was performed according to the method described by Taufiq and Darah (14) in sterilised flat-bottom 96-well microtitre plates.

### Quantification of Biofilm

The quantification of bacterial biofilm was determined according to the procedures described by Taufiq and Darah (14). All experiments were carried out in triplicate. The inhibition activity was expressed in terms of the percentage of biofilm inhibited in comparison to the untreated control biofilm and was calculated according to the following equation:

$$\%\;\text{biofilm inhibition}=\frac{\text{OD growth control}-\text{OD sample}}{\text{OD growth control}}\times 100$$

### Light Microscopy

Light microscopic technique to examine the effects of the fungal extract on the initial and pre-formed biofilms was performed according to Taufiq and Darah (14). The biofilm formation was examined under the light microscope attached with a digital camera (Olympus U-CMAD3).

### Transmission Electron Microscopy

The effects of the fungal extract on MRSA were studied under transmission electron microscopy (TEM) observations. The bacterial suspension was prepared as previously described. A volume of 0.1 mL of bacterial cells was inoculated into each 50 mL Erlenmeyer flask containing 18.9 mL MHB, after which 1.0 mL of the fungal extract was added to each flask to give a final volume of 20 mL and the final concentration in the flask was at the minimum inhibitory concentration (MIC) value. For the control treatment, 1.0 mL of methanol was used to replace the fungal extract and added to the flask. The treated cultures were then incubated at 37 °C for the required incubation time (0 h, 12 h, 24 h and 48 h). The sample preparation for TEM analysis was done following the method described by Taufiq and Darah (10).

### Scanning Electron Microscopy

The effects of the extract on biofilm formation were studied using scanning electron microscopy (SEM). The samples for initial and pre-formed biofilms were prepared according to the previous method. The fixation and dehydration steps as described by Taufiq and Darah (14) were used, each sample was placed on a specimen stub using conductive tape, coated with gold using a sputter coater machine (Fison SC-515, UK) and viewed under a Scanning Electron Microscope (Leica Cambridge, S-360, UK).

### Statistical Analysis

All experimental data were recorded as triplicates. One-way analysis of variance (ANOVA) followed by Duncan’s test was performed, and a significance level of *P* < 0.05 was employed.

## Results

### Structural Degeneration of Bacterial Cells Exposed to the Extract

**Figure 1:**
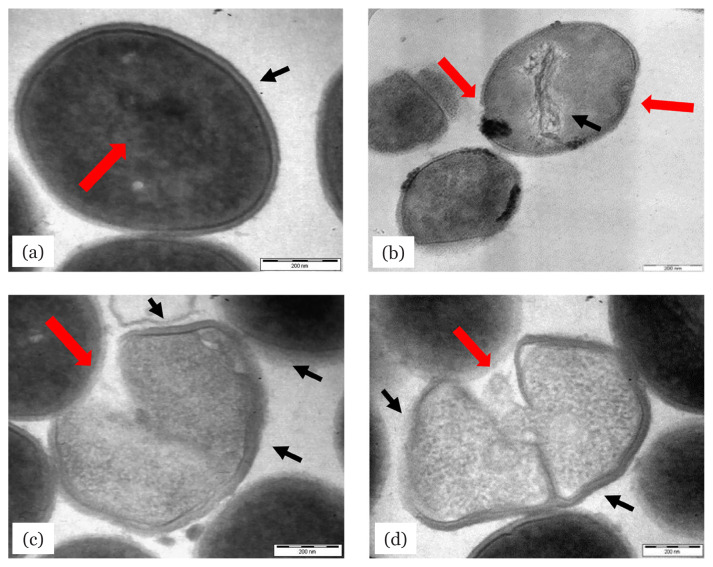
TEM micrographs of MRSA ATCC 33591 treated with 250 μg/mL of *L. pseudotheobromae* IBRL OS-64 ethyl acetate extract at different time exposure time: (a) 0 h [control] (b) 12 h (c) 24 h and (d) 48 h. Scale bars: 200 nm

Figure 1a shows the untreated MRSA ATCC 33591 cells with intact typically spherical shaped cells. The cell was observed to be dense with cytoplasm, organelles and a homologous cell wall (indicated by the red arrow). The typical cell membrane of the Gram-positive bacteria could be seen clearly underneath the cell wall (indicated by black arrow). After 12 h of exposure to the fungal extract, the MRSA ATCC 33591 cells started to disintegrate and alteration within the cells was observed (Figure 1b). This phenomenon was indicated by the disorganising of the cell organelles inside the cytoplasm as highlighted by black arrows. Cell wall disintegration was also observed at this time with the formation of a pit, as indicated by the red arrow. Figure 1c shows the bacterial cells after 24 h exposure to the extract. Some alterations were observed in the internal structures of the bacterial cells, including shrinkage of the cytoplasm and loss of the spherical-shaped structure (indicated by the black arrows). There were also some autolysis and leakage of bacterial cells observed, as indicated by the red arrow, which might lead to the loss of cytoplasm contents. The worst condition was observed on the cell that had been exposed to the fungal extract for 48 h (Figure 1d). The micrograph portrayed the collapse of the bacterial cell, as indicated by the shrunken cell (the site is highlighted by the black arrow), and autolysis which might represent cell leakage (indicated by the red arrow), and loss of organelles in the cytoplasm that would lead to cell death.

### Screening and Qualitative Anti-Biofilm Activity

**Figure 2:**
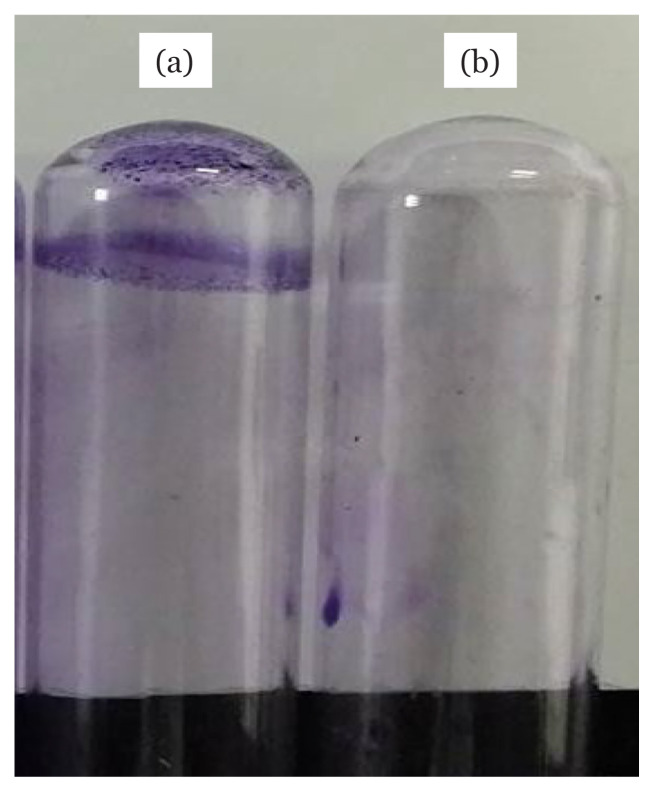
Screening for biofilm producer (a) biofilm producer — MRSA ATCC 33591 (b) non-biofilm producer — MRSA IBRL-1

**Figure 3:**
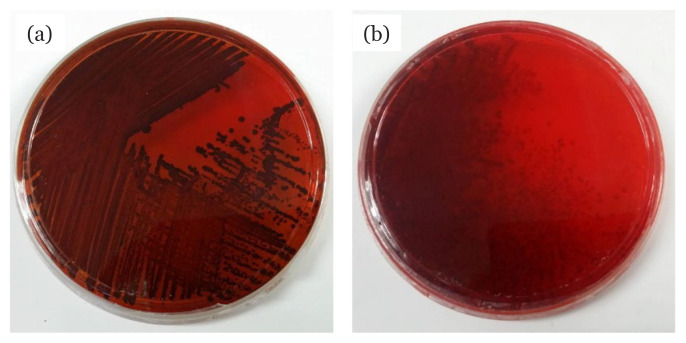
Anti-biofilm activity of fungal crude extract against MRSA ATCC 33591 on CRA: (a) not treated [control] (b) treated

The capability of bacterial strains to form biofilm was determined through CRA method and anti-adherence assays (Table 1). On CRA, the production of black colonies by MRSA ATCC 33591 was observed, indicating the presence of a biofilm-producing strain whereas, for the non-biofilm producer, MRSA IBRL-1, red colonies were formed. Meanwhile, the screening on anti-adherence assay revealed the bacterial strain MRSA ATCC 33591 was a good biofilm producer since the OD reading was in the range of 0.240 and 0.50. Figure 2a shows the formation of biofilm by MRSA ATCC 33591 with a purple stain on the test tube while Figure 2b exhibits the non-biofilm-producing strain, MRSA IBRL-1, with the absence of stain formation. Qualitative anti-biofilm activities of the ethyl acetate extract of *L. pseudotheobromae* IBRL OS-64 against MRSA ATCC 33591 are shown in Table 2. The findings revealed that the extract exhibited moderate anti-adherence activity against the test bacteria on a semi-adherence assay with an inhibition value of 28.23 ± 0.03%. Meanwhile, the qualitative anti-biofilm assay on CRA revealed that the extract possessed fair antibiofilm activity compared to the control (Figure 3).

### Anti-Biofilm Activities of Ethyl Acetate Extract of L. pseudotheobromae IBRL OS-64

**Figure 4:**
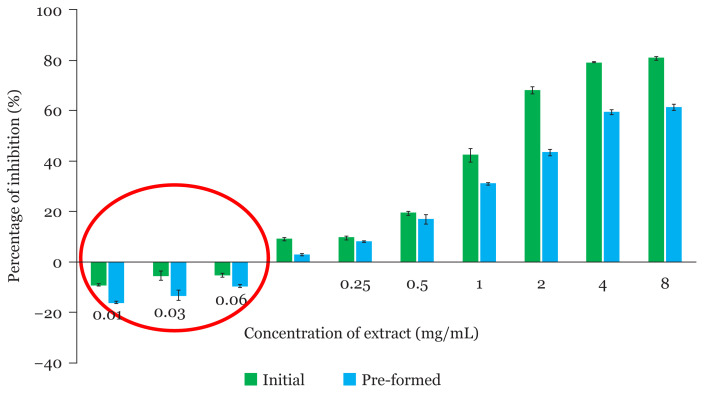
Anti-biofilm activity of ethyl acetate extract of *L. pseudotheobromae* IBRL OS-64 versus the initial and pre-formed biofilm of MRSA ATCC 33591. Red circle shows the antibiotic-induced phenomenon

The percentage of biofilm inhibition of the fungal extract at different concentrations on the initial and pre-formed biofilms of MRSA ATCC 33591 are depicted in Figure 4. The results revealed that the percentage of inhibition increased as the concentration of the fungal extract increased, indicating a concentration-dependent pattern for both the initial and preformed biofilm tests.

In the initial biofilm tests, the highest anti-biofilm activity was observed at an extract concentration of 8.00 mg/mL with 80.82% inhibition. However, the anti-biofilm activity at this concentration was not significantly different from the extract concentration of 4.00 mg/mL, which produced 79.1% inhibition (Duncan’s test, *P* < 0.05). The lowest antibiofilm activity was achieved at a low extract concentration of 0.13 mg/mL with an inhibition value of 8.99%. The anti-biofilm activity of the initial biofilm tests started with a low inhibitory effect, namely, 8.99% at the minimal extract concentration of 0.13 mg/mL and then increased slightly to 9.58% at the extract concentration of 0.25 mg/mL. At the extract concentration of 0.50 mg/mL, the anti-biofilm activity drastically increased with an inhibition value that reached up to 19.39%. The inhibitory effect of the extract at the concentration of 1.00 mg/mL and 2.00 mg/mL rose gradually thereafter with anti-biofilm activities of 42.38% and 68.10%, respectively. These results revealed that the eradication of biofilm was more effective when antibiotic was exposed to the bacterial cells at initial biofilm formation.

In the pre-formed biofilm tests, the extract concentration of 0.13 mg/mL showed the lowest anti-biofilm activity with an inhibition value of 2.88% and the highest activity was achieved at the extract concentration of 8.00 mg/mL, which produced 61.39% of inhibition (Duncan’s test, *P* < 0.05). The fungal extract exhibited biofilm inhibition on both the initial and preformed biofilms and this anti-biofilm property increased as the extract concentration increased. Interestingly, the present findings also indicated an antibiotic-induced phenomenon, as shown by the negative values of inhibition percentage at extremely low concentrations of extracts (0.01 mg/mL, 0.03 mg/mL and 0.06 mg/mL), whereby, the extract is believed to stimulate biofilm formation.

### Observation of the Effects of Ethyl Acetate Extract on Initial and Pre-Formed Biofilm of MRSA Under Light Microscopy

**Figure 5:**
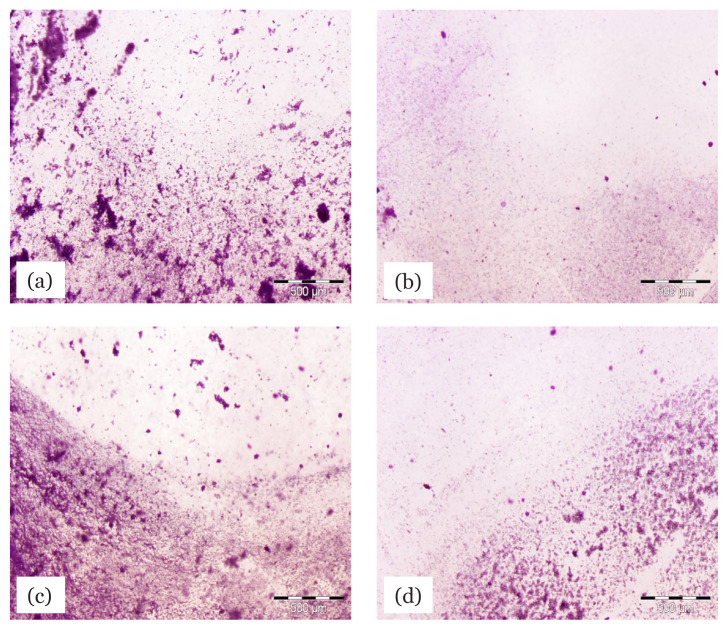
Light microscopy view of MRSA ATCC 33591 biofilm showing the effect of ethyl acetate extract of *L. pseudotheobromae* IBRL OS-64. Figures (a) initial biofilm [control], (b) initial biofilm [treated with fungal extract at concentration of 4 mg/mL], (c) pre-formed biofilm [control] and (d) pre-formed biofilm [treated with fungal extract at concentration of 8 mg/mL]

Figure 5 shows the light microscopy view of MRSA ATCC 33591 biofilms with and without treatment using ethyl acetate extract of *L. pseudotheobromae* IBRL OS-64. Figures 5a and 5b show the initial biofilms which were untreated and treated with 8 mg/mL of extract, respectively, whereas Figures 5c and 5d show the pre-formed biofilms for control and treatment with 8 mg/mL of extract, respectively. In the initial biofilm, the presence of the fungal extract significantly inhibited and decreased biofilm formation. The addition of the fungal extract (Figure 5b) was observed to eradicate the agglutination of microbial matrices seen in the control treatment (Figure 5a). In the pre-formed biofilm (Figure 5c), lower inhibition of biofilm formation was observed when treated with the fungal extract (Figure 5d) compared to the initial biofilm under similar treatment.

### Evaluation of the Effects of Ethyl Acetate Extract on Initial and Pre-Formed Biofilm of MRSA Under Scanning Electron Microscopy

**Figure 6:**
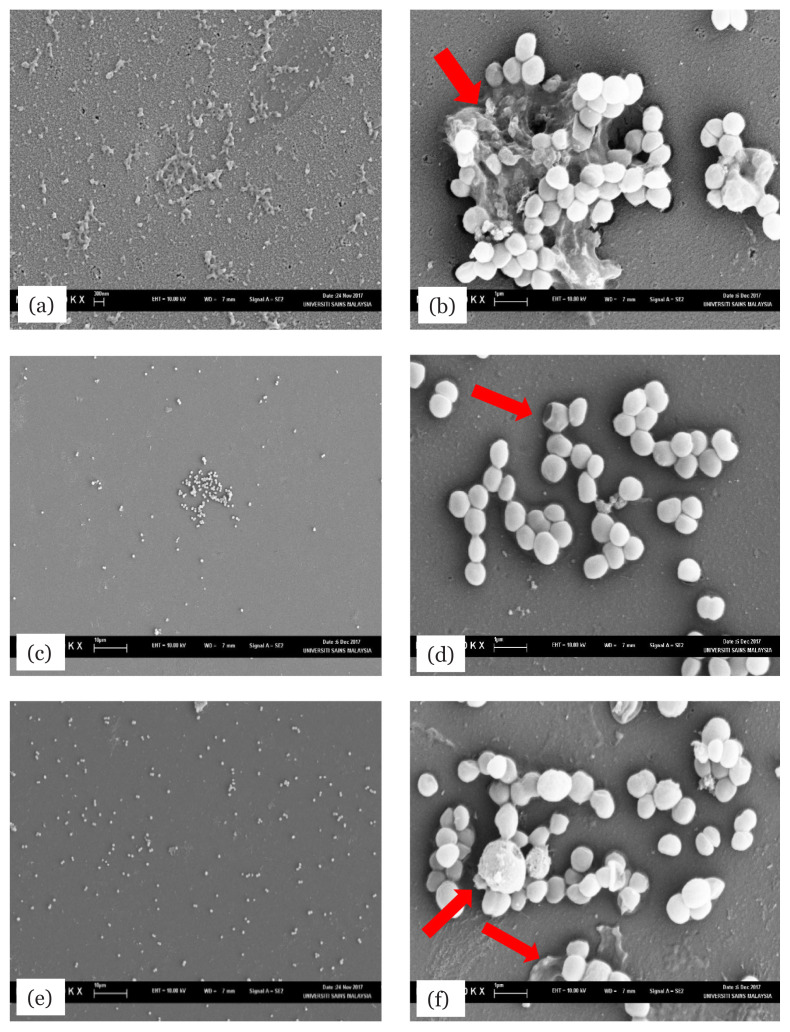
SEM micrographs of MRSA ATCC 33591 biofilm showing the effect of ethyl acetate extract of *L. pseudotheobromae* IBRL OS-64. Figures (a) control [untreated at 1000 ×], (b) control [untreated at 10000 ×] — arrow indicates biofilm formation, (c) initial biofilm [treated with 4 mg/mL of extract at 1000 ×], (d) initial biofilm [treated with 4 mg/mL of extract at 10000 ×] — arrow indicates destruction of bacterial cell, (e) pre-formed biofilm [treated with 4 mg/mL of extract at 1000 ×] and (f) pre-formed biofilm [treated with 4 mg/mL of extract at 10000 ×] — arrows indicate destruction of bacterial cell and elimination of biofilm formation

The effects of ethyl acetate extract of *L. pseudotheobromae* IBRL OS-64 on the initial and pre-formed biofilms of MRSA ATCC 33591 are illustrated in the SEM micrographs in Figure 6. Figure 6a shows the control sample without the fungal extract treatment. Figure 6b shows the same sample at a higher magnification level, where it is seen that the microcolonies were glued together in extracellular polymeric substances (EPS) matrices (red arrow). Figure 6c shows the initial biofilm after treatment with the fungal extract and indicates that both the formation of biofilm and growth of bacterial colony were inhibited. In Figure 6d, disruption of the bacterial cell, cell lysis and their debris (red arrow) could be observed. Figure 6e shows the pre-formed biofilm after exposure to the fungal extract under 1000 × magnification and when a higher magnification was used, reduced biofilm formation could be seen (red arrow) and some of the bacterial cells seemed to have been disrupted and lysed, resulting in uneven cell shape (Figure 6f).

## Discussion

The appearance of community-acquired MRSA is a recent phenomenon that is generating considerable concern worldwide since this type of *S. aureus* could cause severe infections that are difficult to treat in the outpatient setting. This strain can be easily cultured in clinical devices, even after several months, is tolerant of high salt concentrations and possesses relatively heat-resistant characteristics (15). MRSA is a significant pathogen that can cause both nosocomial and community-acquired infections. Thus, a rapid and appropriate antimicrobial therapy is important to control the spread of MRSA strains as they can cause significant morbidity and mortality (16).

The present study revealed that the ethyl acetate extract of *L. pseudotheobromae* isolated from a medicinal plant, *Ocimum sanctum* (*O. sanctum*), exhibited significant antibacterial activity against MRSA. This finding is in agreement with Nurunnabi et al. (17) who reported potential anti-MRSA activity of the endophytic fungus, *Pestalotia* sp., isolated from Sundarbans mangrove plant, *Heritiera fomes*. In the TEM study, the photomicrograph of the thin cross-section revealed that alteration occurred during the MRSA cell lysis process. The fungal extract destroyed the bacterial cells, causing disintegration of the cell wall, formation of pits and leakage of the cytoplasm which resulted in the loss of its contents. A similar observation was reported by Ibrahim et al. (18) who studied the effect of the extract of an endophytic fungus, *Nigrospora sphaerica* CL-OP 30, on MRSA cells. The MRSA cellular damage and cell death observed in the present study could be caused by cell leakages. According to Hossan et al. (19), the bioactive compounds may penetrate and destabilise the cytoplasmic membrane of MRSA resulting in energy and nutrients depletion. Also, the electronegative charge in the bioactive compounds may interfere with the biosynthesis process involving electron transfer and reactions with vital nitrogen components such as proteins, which will then inhibit bacterial cell growth. Even though the mode of action of the fungal extract on the MRSA cells was not studied, but it is believed that the effect of the fungal extract was exerted on the outer layer of the cell membrane, which altered the membrane function, and structure, and permeability.

Biofilms are a consortium of microorganisms enclosed in a matrix that protects microbial growth and allows their survival in a hostile environment (20). Biofilms are composed of attached microbial cells living within an EPS matrix that is made up of extracellular DNA (eDNA), polysaccharides, lipids and proteins. In this study, a preliminary study was employed to screen bacterial biofilm producers. CRA method was performed since it is economical, easy to perform, and involves a simple evaluation of criteria which is based on visual analysis of the colour development of colonies’ that grow on the agar (21). Biofilm producers can produce slime and almost black or black colonies on CRA. Kumar et al. (22) reported the formation of crystalline and black colonies on the agar as a result of the interaction between polysaccharides produced by the test bacteria and the Congo red dye. The present findings revealed that some MRSA strains grew on CRA and formed black colonies while some of them were presence as red colonies. According to Arciola et al. (23), a bacterial strain that grows on the CRA and produces almost black, black, and very black colonies is considered as a biofilm producer, whereas one that grows in colonies with Bordeaux, red and very red colours is a non-biofilm producer.

The present study revealed that the fungal extract can inhibit the initial biofilm more effectively than the pre-formed biofilm. According to Brambilla et al. (24), when the antibiotic was introduced to the bacterial cells at the early stages of growth and before the biofilm is formed, the antibiotic agent can act effectively on the cell growth and thus, inhibit biofilm formation. The capability of antibiotic agents in inhibiting initial biofilm could be of interest especially for combating recalcitrant bacterial infections (25). The findings of the present study also showed an antibiotic-induced phenomenon, as indicated by the negative values of inhibition percentage observed at extremely low concentrations of the fungal extract, namely, 0.01 mg/mL and 0.03 mg/mL. This indicates that the fungal extract could stimulate biofilm formation when present at very low concentrations. This result is in agreement with Ng et al. (26) who reported that induction of MRSA biofilm formation occurred when the bacterial strains were treated with a low concentration of β-lactams antibiotics. They also claimed that the strain that was more sensitive to methicillin demonstrated biofilm induction at a lower level of methicillin as compared to a more resistant strain. According to Kaplan et al. (27), antibiotic-induced biofilm formation was dependent on the secretion of eDNA because an increment of eDNA level in the biofilm matrix was observed at a low dose of methicillin. The excess release of eDNA was contributed by a mutation gene, *atl*, that was responsible for encoding the major *S. aureus* autolysin. Besides that, the eDNA is believed to trigger and enhance biofilm resistance towards antibiotics despite increasing their stability (28).

The current study also clearly revealed that the pre-formed biofilm was more resistant to the inhibitory effects of the fungal extract compared to the initial biofilm. According to Kaplan (29), the higher resistance the pre-formed biofilm might be due to the biofilm dispersal nature of mature bacterial cells that acts as their virulence and survival strategies, which can disperse and disseminate the microcolonies to attach to new surfaces and colonise these surfaces. The addition of antibiotic agents might induce the bacterial biofilm dispersion process in pre-formed biofilm and thus, increase its susceptibility towards antibiotics (30). The results revealed the success of the extract in killing and diminishing microbial cells before they were able to develop a mechanism for biofilm formation. Sandasi et al. (31) reported that good antibiofilm activity may be contributed by significant effect of the extract on the metabolic activity instead of reducing microbial biomass. This phenomenon may be due to the ability of the fungal extract to penetrate the biofilm matrix. Some studies reported that resistance mechanisms of biofilm formation towards the extract can be attributed to several factors, such as low penetration availability of antimicrobial compounds through the microbial matrix, the presence of bacterial communities with different resistance levels, and the persistent presence of microbial cells (32, 33). Xu et al. (34) reported that the EPS matrix of microbial can act as a diffusion barrier to delay and reduce the sensitivity of antibiofilm agents.

In general, there are two suggestions related to the effect of the fungal extract on biofilm formation on the surface of coverslip. Firstly, the extract that is exposed to bacterial cells might kill them before they hit and attach to the coverslip (35). Secondly, the extract might modify the surface of the coverslip and subsequently hinder bacterial growth, adherence, and colonisation on the coverslip (36). The results of this study revealed that the formation of biofilm and bacterial growth were reduced upon exposure to the fungal extract, indicating that the extract possessed significant anti-biofilm activity. The number of free cells of pre-formed biofilm was observed to be slightly higher compared to the initial biofilm, and this may be due to the sensitivity of the bacterial cells towards the presence of antibiotics and the exposure period. Ito et al. (37) observed that the *Escherichia coli* (*E. coli*) cells in the mature biofilm were not completely killed and regrowth occurred after treatment with ampicillin. However, the cells in the young biofilm were completely inhibited and killed by the same antibiotic. They postulated that the resistant mechanism in the mature biofilm of *E. coli* was facilitated with the emergence of resistance sub-populations that were induced by specific phenotypes, responses of gene ribonucleic acid-polymerase sigma (rpOS)-mediated stress and growth rate.

Overall, the fungal crude extract of *L. pseudotheobromae* IBRL OS-64 was proven as a potential antibiofilm agent towards MRSA. There are many possible mechanisms of the killing action by the extract such as anti-quorum sensing, surface modification mechanism and disruption of crucial genes. For instance, Woo et al. (38) reported the effectiveness of antibiofilm activity of the bioactive compounds, dihydrocelastrol and dihydrocelastryl diacetate. They revealed that both these bioactive compounds were able to down-regulated the expression of crucial genes for biofilm formation, such as RNAIII in MRSA, and disturb the gene expression related to α-hemolysin. Although the fungal extract used in this study was observed to possess antibiofilm properties, the mode of action of this extract is still not clear. Therefore, further studies on the mechanism of action of this fungal extract against bacterial cells and their biofilms are required.

## Conclusion

The results showed that the ethyl acetate extract of *L. pseudotheobromae* IBRL OS-64, an endophytic fungus isolated from the leaf of *O. sanctum*, exhibited significant antibacterial and anti-biofilm activities on MRSA cells and could be a good candidate for antibacterial and antibiofilm drugs. The interference of cell membrane permeability and disintegration of cell components could be the probable mechanism of action that causes the bacterial cell lysis. These findings also revealed that the initial biofilm of MRSA was eradicated more effectively by the extract compared to pre-formed biofilm. This may be due to the role of the biofilm as a selective barrier that hinders the antibiotic action, thus requiring a higher concentration of the extract to eliminate the pre-formed biofilm.

## Figures and Tables

**Table 1:** Screening of biofilm forming strain

Test bacteria	Phenotypes on CRA	*OD_570_	Biofilm production
MRSA ATCC 33591	Black	0.476	Producer
MRSA IBRL-1	Red	0.112	Non-producer

Notes:

* Biofilm production: non-producer [OD570 < 0.120], weak producer [0.120 < OD570 < 0.240], producer [0.240 < OD570 < 0.50], high producer [OD570 > 0.50]

**Table 2:** Screening of anti-biofilm activity against test bacterium on CRA and semi-quantitative adherence assay

Test bacterium	CRA observation	Inhibition (%)
MRSA ATCC 33591	++	28.23 ± 0.03

Notes: (+): poor anti-biofilm activity; (++): fair anti-biofilm activity; (+++): good anti-biofilm activity
